# Pharmacokinetics and interspecies scaling of a novel, orally-bioavailable anti-cancer drug, SHetA2

**DOI:** 10.1371/journal.pone.0194046

**Published:** 2018-04-10

**Authors:** Ankur Sharma, Doris Mangiaracina Benbrook, Sukyung Woo

**Affiliations:** 1 Department of Pharmaceutical Sciences, College of Pharmacy, University of Oklahoma Health Sciences Center, Oklahoma City, Oklahoma, United States of America; 2 Department of Obstetrics and Gynecology, College of Medicine, University of Oklahoma Health Sciences Center, Oklahoma City, Oklahoma, United States of America; Northeastern University, UNITED STATES

## Abstract

SHetA2 is a small molecule drug with promising cancer prevention and therapeutic activity and a high preclinical safety profile. The study objectives were to perform interspecies scaling and pharmacokinetic (PK) modeling of SHetA2 for human PK prediction. The PK data obtained from mice, rats, and dogs after intravenous and oral doses were used for simultaneous fitting to PK models. The disposition of SHetA2 was best described by a two-compartment model. The absorption kinetics was well characterized with a first-order absorption model for mice and rats, and a gastrointestinal transit model for dogs. Oral administration of SHetA2 showed a relatively fast absorption in mice, prolonged absorption (i.e., flip-flop kinetics) toward high doses in rats, and an early peak followed by a secondary peak at high doses in dogs. The oral bioavailability was 17.7–19.5% at 20–60 mg/kg doses in mice, <1.6% at 100–2000 mg/kg in rats, and 11.2% at 100 mg/kg decreasing to 3.45% at 400 mg/kg and 1.11% at 1500 mg/kg in dogs. The disposition parameters were well correlated with the body weight for all species using the allometric equation, which predicted values of *CL* (17.3 *L/h*), *V*_*1*_ (36.2 *L*), *V*_*2*_ (68.5 *L*) and *CL*_*D*_ (15.2 *L/h*) for a 70-kg human. The oral absorption rate and bioavailability of SHetA2 was highly dependent on species, doses, formulations, and possibly other factors. The limited bioavailability at high doses was taken into consideration for the suggested first-in-human dose, which was much lower than the dose estimated based on toxicology studies. In summary, the present study provided the PK model for SHetA2 that depicted the disposition and absorption kinetics in preclinical species, and computational tools for human PK prediction.

## Introduction

**Fig 1:**
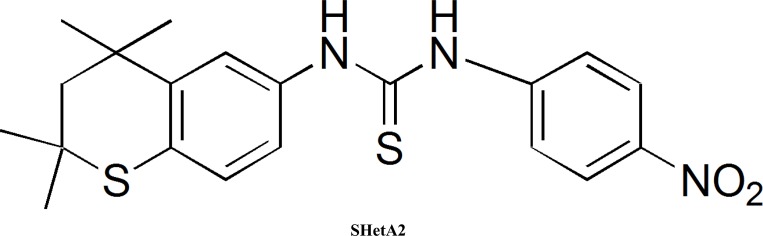
Chemical structure of SHetA2.

The small molecule drug, sulfur heteroarotinoid (SHetA2, Fig 1) [1] is the lead compound of the flexible heteroarotinoids (Flex-Hets), which function independent of retinoic acid receptors and cause potent induction of apoptosis in cancer cells without harming normal cells [2]. SHetA2 induces G1 cell arrest and apoptosis in human ovarian cancer cells, regardless of histology, with an IC_50_ of ~0.37–4.6 *μM* for growth inhibition in the National Cancer Institute 60 cell line screen [3]. However, normal and benign epithelial cells only undergo G1 cell cycle arrest and remain resistant to these apoptotic effects [4, 5]. The mechanism of this differential effect appears to occur through the SHetA2-binding proteins Heat Shock Protein A (HSPA9, HSPA8, and HSPA5) [6]. Down-stream effects of SHetA2 binding to these proteins lead to degradation of cyclin D1 in both normal and cancer cells and Bcl-2 and Bcl-xl in cancer cells only. SHetA2 induces degradation of cyclin D1, which plays a major role in G1 cell cycle arrest induced by SHetA2 in both ovarian cancer and normal cells [7]. Degradation of Bcl-2 and Bcl-xl is associated with apoptosis in SHetA2-treated ovarian cancer cells, while normal cells remain resistant to these drug effects [8].

Oral administration of a 10 mg/kg dose of SHetA2 led to significant tumor growth inhibition in a human ovarian cancer xenograft model [3]. SHetA2 also demonstrated antitumor activity at a dose range of 30–60 mg/kg in kidney cancer [5] and in colon and intestinal cancer in the APC^min^ mouse model [1]. Extensive preclinical testing revealed that SHetA2 lacked mutagenicity, carcinogenicity, and teratogenicity [9, 10]. SHetA2 has a wide therapeutic window with a No-Observed-Adverse-Effect level (NOAEL) of >1500 mg/kg/day in a 28-day dog toxicity study [11]. These tumor-selective activities and broad safety profile make SHetA2 an ideal drug for cancer prevention [6].

SHetA2 is highly hydrophobic and has low GI absorption with low bioavailability (<1%) in rats [11]. However, the bioavailability in dogs was improved with a formulation of SHetA2 suspended in 30% aqueous Solutol HS 15 (2-hydroxyethyl 12-hydroxyoctadecanoate) [11], a non-ionic, non-toxic solubilizer and emulsifier used for preparing stable formulations. To demonstrate whether oral administration of SHetA2 can achieve physiological concentrations sufficient for target modulation, a phase 0 clinical study has been proposed. Phase 0 trials represent a means of accelerating drug development to assess the feasibility for further clinical development of investigational agents prior to traditional phase 1 trials [12]. These trials are designed at doses that result in limited human exposure with no diagnostic or therapeutic intent, but instead aim to study pharmacokinetic properties in humans and/or concentrations that cause target modulation [12]. The objectives of the present study were to characterize the preclinical pharmacokinetics of SHetA2 using interspecies scaling and PK modeling and to predict human PK to support the Phase 0 study design.

## Materials and methods

### Data collection

The pharmacokinetic data of SHetA2 after intravenous (IV) and oral administration at various doses were obtained from the literature and study reports for three species: mice [13], rats [11], and dogs [11]. The data from IV administration of 5 mg/kg in dogs were obtained from a pilot PK and tolerability study in beagle dogs (N01-CN-43306 Work assignment 21, NCI). Table 1 summarizes the details of drug doses/formulations and animal body weights. CD2F1 mice (20.0–27.8 g) received 20 and 60 mg/kg oral doses of SHetA2 by gavage in 100 μl of dosing solution, and 20 mg/kg IV dose through the tail vein in 100 μl of dosing solution adjusted by body weight. The animals were not fasted. Blood was collected by cardiac puncture from individual animals at pre-dose and 0.08, 0.15, 0.25, 0.5, 1, 2, 3, 4, 6, 8, 12, 18, 24, 36, 48, and 60 h after IV dosing. Blood samples were also collected by cardiac puncture at pre-dose and 0.25, 0.5, 1, 2, 3, 4, 6, 8, 12, 18, 24, 36, and 48 h after oral administration. The PK data were presented as mean ± standard deviation (sd). Crl:CD (SD) rats (260–347 g) received SHetA2 solution intravenously as a single 5 mg/kg dose, and in oral suspension at 100, 500, or 2000 mg/kg doses, by gavage at a dosing volume of 10 mL/kg/day, daily for 28 days. Serial blood from non-fasted rats was collected by a retro-orbital puncture at 0, 0.08, 0.25, 0.5, 1, 2, 4, and 6 h after IV dosing. Similarly, serial blood samples were also obtained on week 4/5 at 0, 0.5, 1, 2, 4, 6, 8, and 24 h after oral administration for PK evaluation [11]. The PK data for rats were digitized using GetData Graph Digitizer (version 2.26.0.20).

**Table 1:** Summary of SHetA2 pharmacokinetic studies in mice, rats, and dogs.

Species	Routes	Dose (mg/kg)	Formulation	Body Weights (kg)	Reference
**Mouse**	PO	20	Sesame oil	0.020–0.028 (female)	[13]
		60			
	IV	20	PEG400:Ethanol:Saline (57.1%: 14.3%: 28.6%)		
**Rat**	PO	100	1% Methylcellulose/ 0.2% Tween 80	0.260–0.347	[11]
		500			
		2000			
	IV	5	PEG400:Ethanol:Saline (57.1%: 14.3%: 28.6%)		
**Dog**	PO	100	30% Aqueous Solutol HS 15	9.0–11.2; 6.9–8.4 (female)	N01-CN-43306 NCI Toxicological report
		400			
		1500			
	IV	5	Not reported	6.4–7.0	

SHetA2 was administered to beagle dogs (6.4–11.2 kg) as an oral suspension at a dose of 100, 400, or 1500 mg/kg (dosing volume of 5 mL/kg/day), and as an intravenous solution at a dose of 5 mg/kg. Blood from non-fasted dogs was collected from the jugular vein at pre-dose and 1, 2, 3, 4, 6, 9, and 24 h after oral administration [11]. Blood samples were also collected at 0, 0.08, 0.25, 0.5, 1, 2, 4, 8, and 24 h after IV administration (N01-CN-43306 Toxicological report, NCI). The PK data for dogs after oral administration represent mean ± sd and after IV administration represent mean (n = 2) only.

### Plasma SHetA2 quantification

Plasma concentrations of SHetA2 in mouse plasma were quantified using solid-phase extraction and high performance liquid chromatography-UV detection (341 nm) with a lower limit of quantification of 10 ng/ml, as described elsewhere [13]. Plasma concentrations of SHetA2 in rat and dog plasma were determined with high performance liquid chromatography and tandem mass spectrometry (LC/MS/MS) using a gradient method using 0.01% trifluoroacetic acid (TFA) in water (mobile phase A) and 0.01% TFA in acetonitrile (mobile phase B). Briefly, samples were mixed with an ice-cold internal standard and ice-cold acetonitrile, and vortexed thoroughly to ensure good mixing and extraction. After the centrifugation, the upper organic layer was transferred and mixed with ice-cold mobile phase A. The mixture was centrifuged and placed into the autosampler (4°C) for the analysis. The analytical method was validated with a lower limit of quantification of 10 ng/mL, intra- and inter-day accuracy within 80–120%, and precision within 15% (N01-CN-43306 Toxicological report, NCI). Plasma samples (in lithium heparin) were stored at -80 (±20)°C until analysis and were thawed on wet ice to avoid any degradation of the drug.

### Model development

**Fig 2:**
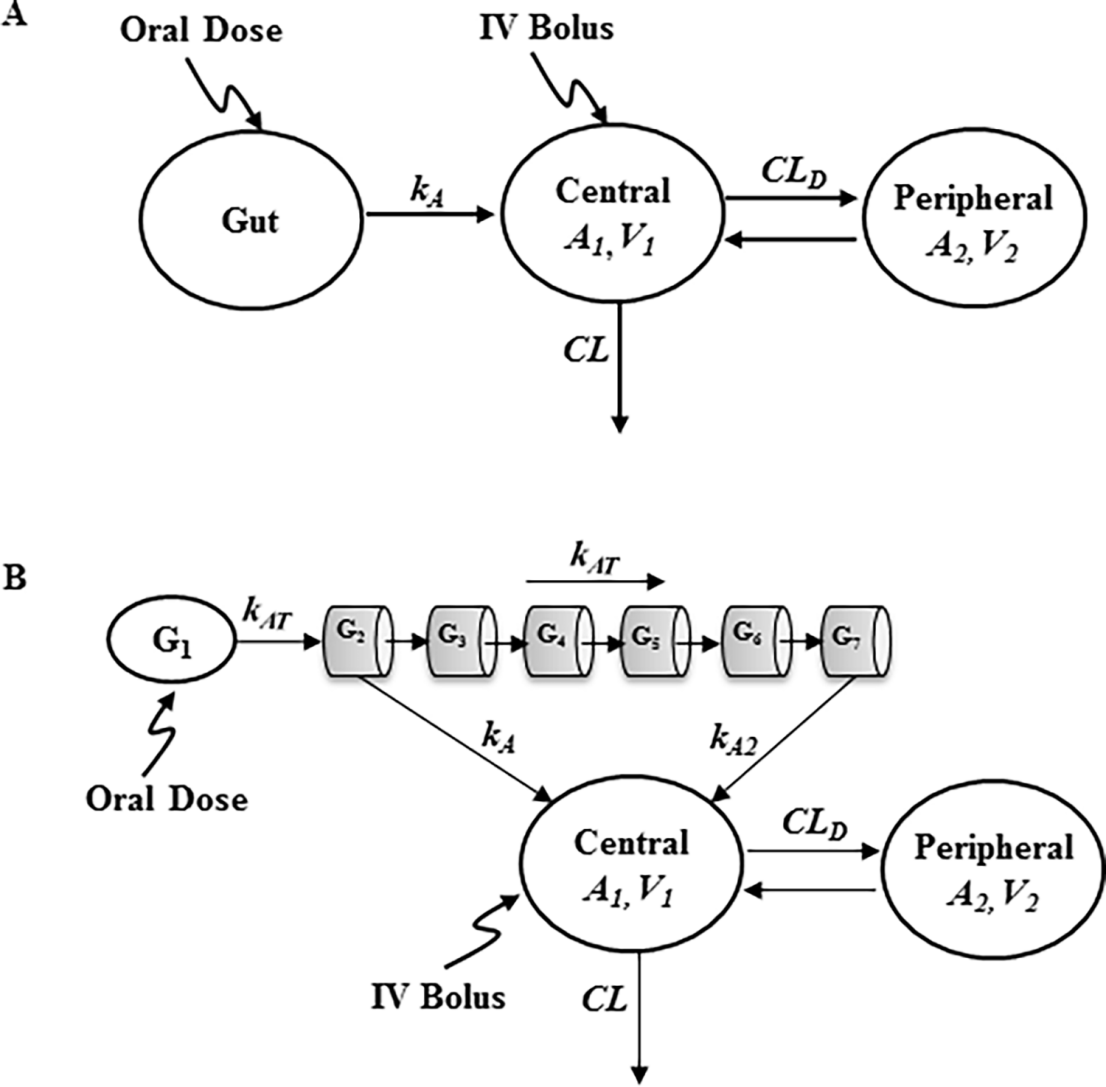
A two-compartment PK model with a first-order absorption for SHetA2 in mice and rats (A) and with a gastrointestinal transit absorption in dogs that accounts for SHetA2 transit through serial compartments after oral administration in dogs (B).

A two-compartment model (Fig 2) was used to characterize the concentration-time profiles, which showed a biexponential decline, following IV bolus administration of SHetA2 in mice, rats, and dogs. From the central compartment, SHetA2 is eliminated by a first-order process and distributes into the peripheral compartment. For oral administration of SHetA2 in mice and rats, an absorption compartment was added (Fig 2A). The differential equations and initial conditions specifying the drug disposition and oral absorption in mice and rats (Fig 2A) are shown:

$$\frac{dA_{1}}{dt}=k_{A}∙G-\left(\frac{{CL}_{D}}{V_{1}}+\frac{CL}{V_{1}}\right)∙A_{1}+\frac{{CL}_{D}}{V_{2}}∙A_{2};\;A_{1}(0)=0$$ (1)

$$\frac{dA_{2}}{dt}=\frac{{CL}_{D}}{V_{1}}∙A_{1}-\frac{{CL}_{D}}{V_{2}}∙A_{2};\;A_{2}(0)=0$$ (2)

$$\frac{dG}{dt}=-k_{A}∙G;\;G(0)=Dose∙F$$ (3)

where *A*_*1*_ and *A*_*2*_ represent the amount of SHetA2 in the central and peripheral compartment, and G represents the amount of SHetA2 in the gut absorption compartment. The two-compartment model was parameterized with clearance (*CL*), volume of distribution in the central (*V*_*1*_) and peripheral (*V*_*2*_) compartment, and distribution clearance (*CL*_*D*_) between compartments. *k*_*A*_ is the first-order absorption rate constant and *F* is oral bioavailability.

For oral administration in dogs, the absorption kinetics were characterized using a transit compartment model that assumed variability in absorption through different segments of the gastrointestinal tract, which was partitioned into seven serial compartments (*G*_*1*_*-G*_*7*_; Fig 2B), similar to the advanced compartmental absorption and transit (ACAT) model [14, 15]. In this model, we assumed (a) the drug undergoes transit through the intestines with the first-order transit rate constant (*k*_*AT*_), which was assumed to be same through all compartments; (b) SHetA2 absorption from the first compartment (i.e., stomach) is insignificant; and (c) the absorption only occurs from the second (G_2_) and last (G_7_) segments with two different absorption rate constants (*k*_*A*_ and *k*_*A2*_), with no absorption from segments 3–6. The differential equations and initial conditions specifying the drug disposition and oral absorption in dogs (Fig 2B) are expressed as:

$$\frac{dA_{1}}{dt}=k_{A}∙G_{2}+k_{A2}∙G_{7}+\frac{{CL}_{D}}{V_{2}}∙A_{2}-\left(\frac{{CL}_{D}}{V_{1}}+\frac{CL}{V_{1}}\right)∙A_{1};\;A_{1}(0)=0$$ (4)

$$\frac{dA_{2}}{dt}=\frac{{CL}_{D}}{V_{1}}∙A_{1}-\frac{{CL}_{D}}{V_{2}}∙A_{2};\;A_{2}(0)=0$$ (5)

$$\frac{dG_{1}}{dt}=-k_{AT}∙G_{1};\;G_{1}(0)=Dose∙F$$ (6)

$$\frac{dG_{2}}{dt}=k_{AT}∙G_{1}-(k_{A}+k_{AT})∙G_{2};\;G_{2}(0)=0$$ (7)

$$\frac{dG_{N}}{dt}=k_{AT}∙(G_{\left(N-1\right)}-G_{N});\;G_{N}(0)=0\;(N=3-6)$$ (8)

$$\frac{dG_{7}}{dt}=k_{AT}∙G_{6}-k_{A2}∙G_{7};\;G_{7}(0)=0$$ (9)

where *G*_*1*_*-G*_*7*_ represent the amount of SHetA2 in gastrointestinal absorption compartments. *k*_*A*_ and *k*_*A2*_ represent the first-order absorption rate constant from the second (G_2_) and last (G_7_) compartments, respectively.

The plasma concentration-time data from both IV and oral routes were fitted simultaneously with Eqs 1–3 (Fig 2A) to obtain the disposition (*CL*, *V*_*1*_, *V*_*2*_, *CL*_*D*_) and absorption parameters (*k*_*A*_ and *F*) for mice and rats. Similarly, the parameters for disposition and gastrointestinal transit absorption process (*k*_*A*_, *k*_*A2*_, *k*_*AT*_, and *F*) were obtained by simultaneously fitting the IV and oral data from dogs with Eqs 4–9 (Fig 2B). The units of parameters are *L/h* for *CL* and *CL*_*D*_; *L* for *V*_*1*_ and *V*_*2*_; and *h*^*-1*^ for all first-order rate constants (e.g., *k*_*A*_, *k*_*A2*_, *k*_*AT*_).

Phoenix® WinNonlin® version 6.4 (Certara USA, Inc., Princeton, NJ) was used for noncompartmental analysis and PK modeling. The noncompartmental parameters included the area under the plasma-concentration curve (*AUC*_*0-inf*_), maximum concentration (*C*_*max*_), time of *C*_*max*_ (*T*_*max*_), and terminal half-life (*t*_*1/2*_). All computer fitting was performed using the minimization algorithm of Gauss-Newton (Levenberg and Hartley) and the weighting scheme of 1/y^2^. Model performance evaluation and selection was based on the objective function, Akaike Information Criterion (AIC), the precision of the parameter estimates, and standard visual inspection of the overall goodness-of-fits.

### Allometric scaling

Allometric scaling is based on the concept that anatomical, physiological, and biochemical variables of different mammals can be scaled across species with respect to body weight [16]. Allometric scaling was applied to the disposition parameters, including clearance (*CL*), central (*V*_*1*_) and peripheral (*V*_*2*_) distributional volumes, and distributional clearance (*CL*_*D*_), obtained from simultaneous fitting of oral and IV plasma concentration-time data for each species. The disposition parameters were plotted against the body weights (BW) on a log-log scale, and human disposition parameters were scaled corresponding to body weight of 70 kg by performing least-squares linear regression to the power-based simple allometric equation, *P* = *a* • *BW^b^* where, *P* = PK parameter of interest, *a* = allometric coefficient, and *b* = allometric exponent.

A previous in vitro study reported extensive non-enzymatic hydrolysis of SHetA2 in plasma from mice, rats, dogs, and humans at body temperature [13]. SHetA2 also appears to undergo hepatic metabolism, as metabolic products of SHetA2, GSH adducts, and mono- and dihydroxylated SHetA2, have been identified in rat and human liver microsomes and in rat plasma [17]. In general, simple allometry works well for drugs that are mainly renally eliminated, but prediction error is high for hepatically eliminated small molecule drugs because of high cross-species variability in hepatic metabolism. Several studies have shown that modified scaling methods with a correction of maximum life-span potential (MLP) or brain weight (BRW) improve the accuracy of human CL prediction [18–20], as longevity is frequently inversely correlated with hepatic cytochrome P450 drug oxidation rates [18, 21]. Therefore, in addition to simple allometry, we applied the MLP correction in the prediction of SHetA2 clearance in humans using *CL* ∙ *MLP* = *a* • *BW^b^* [20].

### First-in-human dose

The first-in-human dose was designed based on the original recommendation [12] for calculation of clinical start dose. This states that the starting clinical dose should be less than 1/50 of the NOAEL from the 2-week toxicology study in the most sensitive species. The maximum clinical dose can be the lowest of the following: (1) ¼ of the 2-week rodent NOAEL; (2) up to ½ of the AUC at the NOAEL in the 2-week rodent study, or the AUC in the dog at the rat NOAEL, whichever is lower; (3) the dose that produces a pharmacologic and/or pharmacodynamic response or at which target modulation is observed in the clinical trial; and (4) observation of an adverse clinical response. Previous studies [11] have shown that the NOAEL for rodent and non-rodent species varied from 500–1500 mg/kg/day. Hence, theoretically, the clinical starting dose may range from 10–30 mg/kg/day in humans. The guidelines also suggest considering a dose at which saturation of drug absorption occurs and that causes no toxicity [22]. Since SHetA2 has limited aqueous solubility, which may contribute to its low, dose-dependent bioavailability, we compared the bioavailability of SHetA2 with respect to doses across species and estimated the dose at which saturation in absorption may occur. Such a dose may be considered further in deciding the first-in-human dose.

### Simulation of human pharmacokinetics

To assess the impact of variability associated with physiological and drug-specific parameters on the oral PK profile of SHetA2 in humans, we performed virtual clinical trial simulations using GastroPlus version 9.5 (Simulations Plus, Inc., Lancaster, CA), a physiologically-based pharmacokinetic (PBPK) modeling software. The oral pharmacokinetics of SHetA2 were simulated by linking a two-compartment drug disposition model to the Advanced Compartmental Absorption and Transit (ACAT) model in GastroPlus. This physiological absorption model accounts for pH, transit time, lengths and radii of each gastrointestinal compartment, plasma protein binding, stomach volume, hepatic blood flow rate, and gut enzyme and transporter distribution. The input data for SHetA2 compound included (a) disposition PK parameters from the allometically-scaled human values of CL, *V*_*1*_, *V*_*2*_, and *CL*_*D*_; (b) SHetA2 doses in mg; and (c) physicochemical properties, including molecular structure (Fig 1), experimentally calculated log P value of 4.23 [23], permeability [24], and experimentally calculated solubility of 19.9 μg/mL in simulated gastrointestinal fluid. For the population demographic, physiological, and other variables, we used the default values and variability (e.g., 40% for CL and 20% for *V*_*1*_) provided in the program. The population simulations were conducted for 100 healthy subjects with the suggested first-in-human doses. The 5th and 95th percentiles of the simulated concentration time profiles were represented by the 90% probability contour.

## Results

### Pharmacokinetics of SHetA2

**Fig 3:**
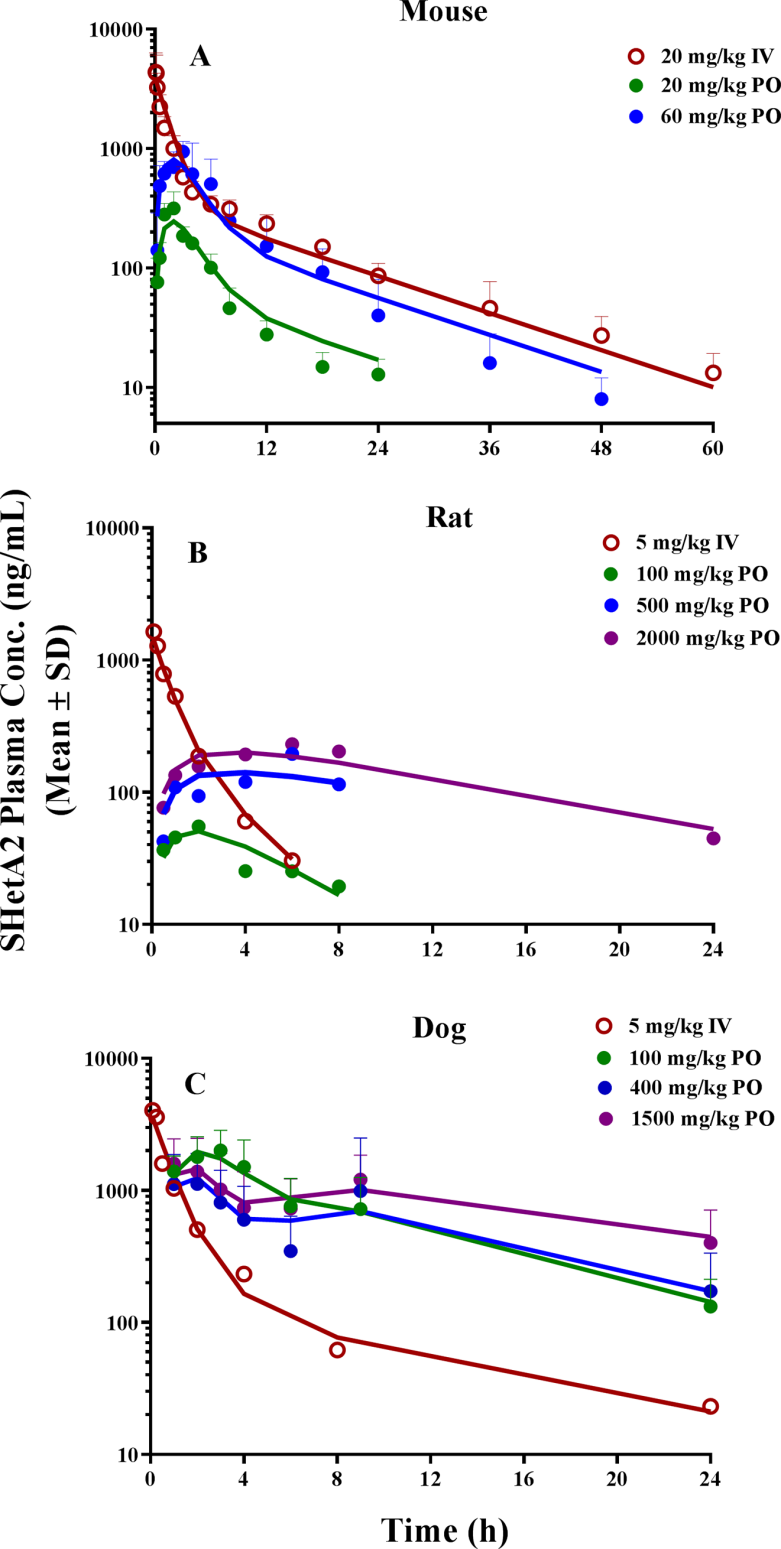
The plasma concentration-time profiles of SHetA2 following (A) 20 mg/kg IV dose and oral dose of 20 and 60 mg/kg in in mice (n = 3/dose), (B) 5 mg/kg IV dose and oral dose of 100, 500, and 2000 mg/kg in rats (n = 3/dose), and (C) 5 mg/kg IV dose (n = 2) and oral dose of 100, 400, and 1500 mg/kg (n = 4/dose) in dogs. Solid lines indicate the model-predicted values after simultaneous fitting of IV and oral PK data to the proposed PK models in each species. Symbols indicate the observed plasma concentrations (mean ± sd).

Fig 3 shows the observed PK data after IV and oral doses in mice, rats, and dogs. The plasma concentration-time profiles after IV administration in all species exhibited a biexponential decline and were best described by a two-compartment model. For each species, the IV and oral data were simultaneously fitted to a two-compartment model with either a first-order absorption model (mouse and rat; Fig 2A) or a gastrointestinal transit absorption model (dog; Fig 2B). The estimated PK parameters are summarized in Tables 2 and 3. SHetA2 appeared to be readily distributed into peripheral tissues, as indicated by a high extent of the volume of distribution. In mice, plasma concentrations reached the peak at 2 h after oral administration and declined in parallel with the terminal slope of the IV profile, with elimination half-lives of 7–11 h. The elimination half-life of SHetA2 after IV administration in mice was much longer than it was in rats (1.16 h) (Table 2). This disposition pattern in non-tumor-bearing mice was also different from what our group observed for SHetA2 in tumor-bearing mice (*t*_*1/2*_ = 4.5 h) [23]. The oral bioavailability was estimated to be 17.7% and 19.5% at 20 and 60 mg/kg.

**Table 2:** Pharmacokinetic parameters for mice, rats, and dogs obtained by the noncompartmental analysis.

*Dose (mg/kg)*	Mouse			Rat				Dog			
	IV	Oral		IV	Oral			IV	Oral		
	20	20	60	5	100	500	2000	5	100	400	1500
***AUC***_***0-inf***_ ***(ng·h/mL)***	10942	1801	6875	1583	542	1413	3586	4885	16813	14593	25082
***C***_***max***_ ***(ng/mL)***	4370	316	944	1642	55	195	231	4034	2004	1121	1595
***T***_***max***_ ***(h)***	0.1	2.0	3.0	0.1	2.0	6.0	6.0	0.1	3.0	2.0	1.0
***t***_***1/2***_ ***(h)***	11.5	10.8	7.02	1.16	10.4	NA	7.49	6.93	6.77	5.94	9.43

**Table 3:** Pharmacokinetic parameter estimates for mice, rats, and dogs obtained by simultaneously fitting oral and IV data to compartmental PK models (Fig 2).

			Estimate (CV%)					
Parameters	Mouse		Rat			Dog		
***V***_***1***_ **(L)**	0.121 (16)		0.969 (18)			8.53 (18)		
***CL***_***D***_ **(L/h)**	0.0323 (20)		0.286 (70)			3.24 (29)		
***V***_***2***_ **(L)**	0.282 (14)		0.531 (48)			22.5 (26)		
***CL* (L/h)**	0.0421 (8)		0.916 (10)			6.39 (10)		
			***Dose (mg/kg)***					
	**20**	**60**		**100**	**500**	**2000**		**100**	**400**	**1500**
***F* (%)**	17.7 (12)	19.5 (12)		1.03 (14)	1.57 (19)	0.560 (16)		11.2 (16)	3.45 (18)	1.11 (18)
***k***_***A***_ **(h**^**-1**^**)**^**a**^	0.539 (19)		0.0755 (17)			1.12 (43)		0.411 (21)
***k***_***A2***_ **(h**^**-1**^**)**^**b**^	-			-		0.929 (281)	0.105 (28)	0.0898 (44)
***k***_***AT***_ **(h**^**-1**^**)**^**c**^	-			-		0.532 (35)	1.11 (25)	1.25 (31)

^a^ first-order absorption rate constant from the absorption compartment for mice and rats (Fig 2A) and G2 compartment for dogs (Fig 2B)

^b^ first-order absorption rate constant from the G7 compartment

^c^ first-order intestinal transit rate constant

For oral administration in rats, the prolonged absorption kinetics were apparent at higher doses (500 and 2000 mg/kg), as evidenced by the estimated absorption rate constant (*k*_*A*_) of 0.0755 h^-1^ and a much slower decline of drug concentration following the oral dose compared with the IV administration (i.e., flip-flop kinetics). The peak concentrations were achieved in 2 h at 100 mg/kg and later around 6 h at higher doses of 500 and 2000 mg/kg. The oral bioavailability in rats was low, ranging from 0.56–1.6% at all studied doses, and the increase in overall systemic exposure (C_max_ and AUC) was less than proportional at 2000 mg/kg (Table 3).

In dogs, the oral absorption was relatively fast, reaching the peak concentration within 1–3 h after dosing. The increases in C_max_ and AUC were less than proportional with increasing doses (Table 2), indicating the nonlinear PK. The mean C_max_ observed at two high doses of 400 and 1500 mg/kg was smaller than that at 100 mg/kg. Oral administration of 400 and 1500 mg/kg displayed second peak at around 9 h that was absent from the IV profile, but the second peak did not manifest at 100 mg/kg. This absorption phenomenon was characterized using a transit compartment model with variability in absorption through the gastrointestinal tract (Fig 2B). In this model, drug movement along the intestine was modeled by a first-order process (*k*_*AT*_) through seven transit compartments and drug absorption by two different first-order rate constants (*k*_*A*_ and *k*_*A2*_) occurring from the G2 and G7 compartments. Multiple transit compartments were needed to capture a significant time delay between initial (at 1–3 h) and second (at 9 h) peaks. An appropriate number of transit compartments was selected based on trial and error by comparing the standard model selection criteria. The magnitude and time of initial and/or second peak occurrences varied by doses after oral administration, and bioavailability (*F*) was markedly reduced with increasing doses. This required the estimation of the rate and extent of absorption kinetic parameters varying at different doses; those parameters included *F*, *k*_*AT*_, *k*_*A*_, and *k*_*A2*_.

Our results indicated that the drug absorption from the G2 compartment (*k*_*A*_) was much slower at higher doses than at low doses (0.411 vs. 1.12 *h*^*-1*^), which contributed to the first peak being lower and delayed at higher doses. The second peak, which occurred at around 9 h, was depicted by a combination of varying transit constant (*k*_*AT*_; 0.532–1.25 *h*^*-1*^) and absorption rate constant from the G7 compartment (*k*_*A2*_; 0.929–0.0898 *h*^*-1*^) at different doses (Table 3). Our proposed model captured the observed plasma concentration-time profiles reasonably well and exhibited superior performance to the conventional single first-order absorption model. Although a Michaelis-Menten type of nonlinear absorption kinetic model could have been explored, we chose to estimate a separate set of absorption parameters for each dose because, considering a wide range of doses studied in rats and dogs (100–1500 mg/kg), three dose levels were not enough to accurately characterize the nonlinearity. The oral bioavailability of the SHetA2 formulation in dogs was reduced from 11.2% at 100 mg/kg to 3.45% at 400 mg/kg to 1.11% at 1500 mg/kg.

**Fig 4:**
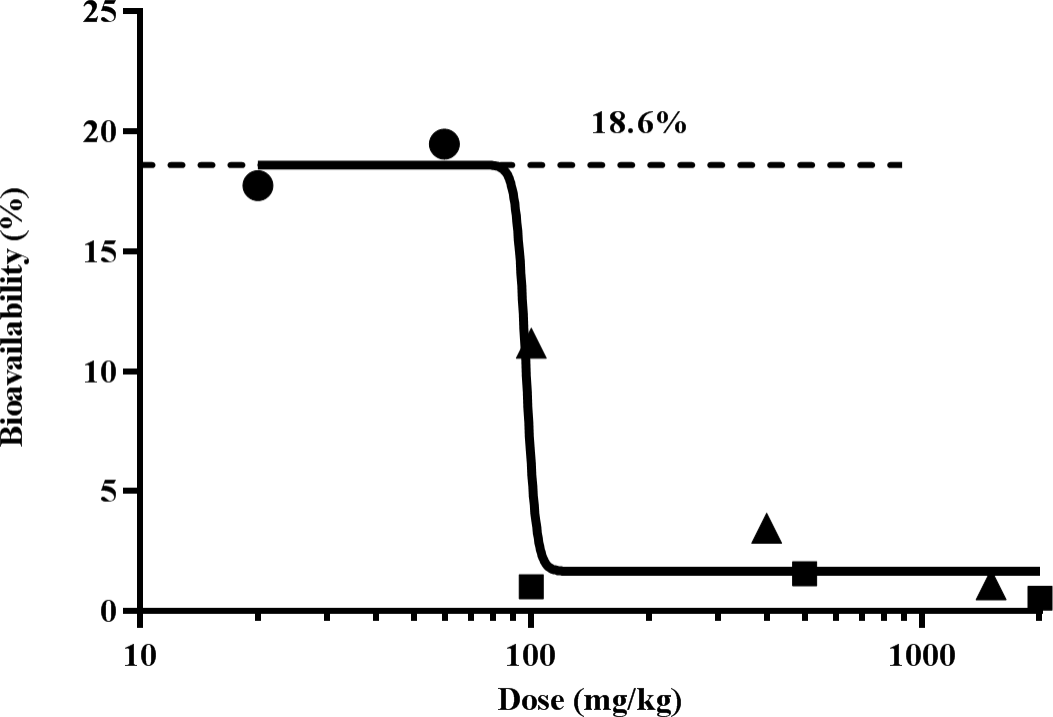
Log dose vs. oral bioavailability among mice (●), rats (■), and dogs (▲) showing the maximum extent of absorption of 18.6% at doses <100 mg/kg.

Fig 4 shows the comparison of oral bioavailability with respect to dose among mice, rats, and dogs. The oral bioavailability (18.6%) in mice after 20–60 mg/kg was the highest among all preclinical studies. In dogs, bioavailability dose-dependently decreased with increasing doses. In a separate experiment, the solubility of SHetA2 was studied in simulated gastrointestinal fluid and was found to be 2% w/v (19.9 μg/mL). Considering the limited solubility of SHetA2, one possibility could be that absorption saturation reached <100 mg/kg dose. Compared with mice and dogs, oral bioavailability in rats was generally low at all doses (*F*<1.6%), which could be attributed to several factors, including different formulation used in rats, species differences in absorption through the gastro-intestinal tract and first-pass metabolism, and different dietary habits among species.

### Allometric scaling

**Fig 5:**
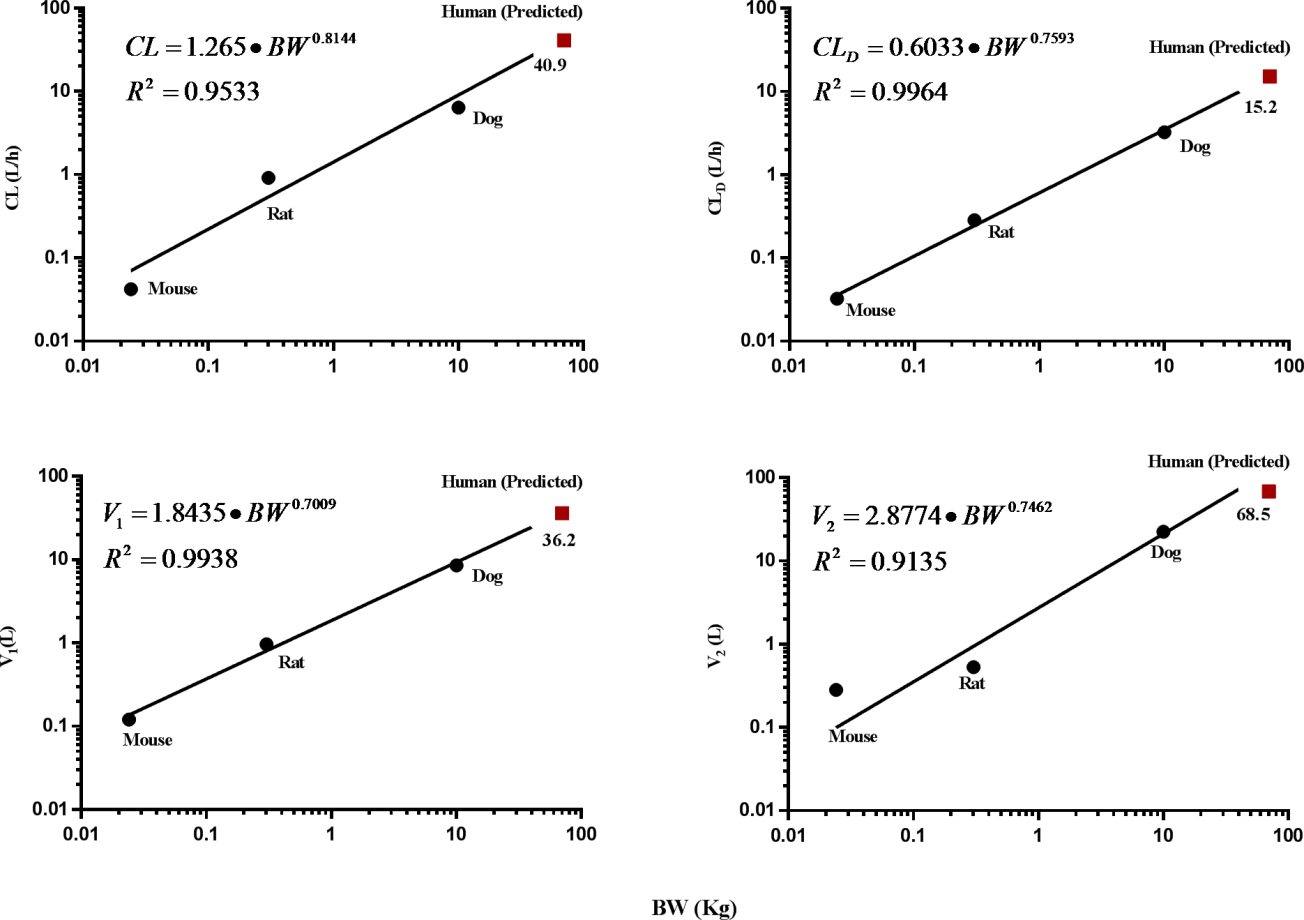
Log-log representation of the allometric relationship in various pharmacokinetic parameters: clearance (A), distribution clearance (B), and central (C) and peripheral (D) volume of distribution.

Interspecies scaling was performed on the disposition parameters of clearance (*CL*), central (*V*_*1*_) and peripheral (*V*_*2*_) distributional volumes, and distributional clearance (*CL*_*D*_; Table 3). Each of the four PK parameters were well correlated with the body weights for all species (*R*^2^ = 0.91 − 0.99; Fig 5). These disposition parameters were then extrapolated for a 70-kg human. In addition to the simple allometry, we also applied the MLP-based correction method to predict clearance as SHetA2 undergoes degradation in plasma and hepatic metabolism. The predicted human clearance ranged from 17.3 L/h (MLP-based) to 41.0 L/h (simple allometry).

### Prediction of human pharmacokinetics

Based on the limited oral absorption observed at higher doses (Fig 4), we chose 100 mg/kg as a new NOAEL, not the highest dose of 1500 mg/kg from the toxicology studies, to find a first-in-human dose. One-fiftieth of the new NOAEL [22] gave the starting minimum dose of 2 mg/kg for the planned Phase 0 clinical trial. Doses can be escalated from 2 mg/kg to a point at which the peak plasma concentration (C_max_) reaches 4 μM (equivalent to 1600 ng/ml), the concentration that is sufficient to induce G1 cell cycle arrest within 24 hours and causes target modulation within 4 hours.

**Fig 6:**
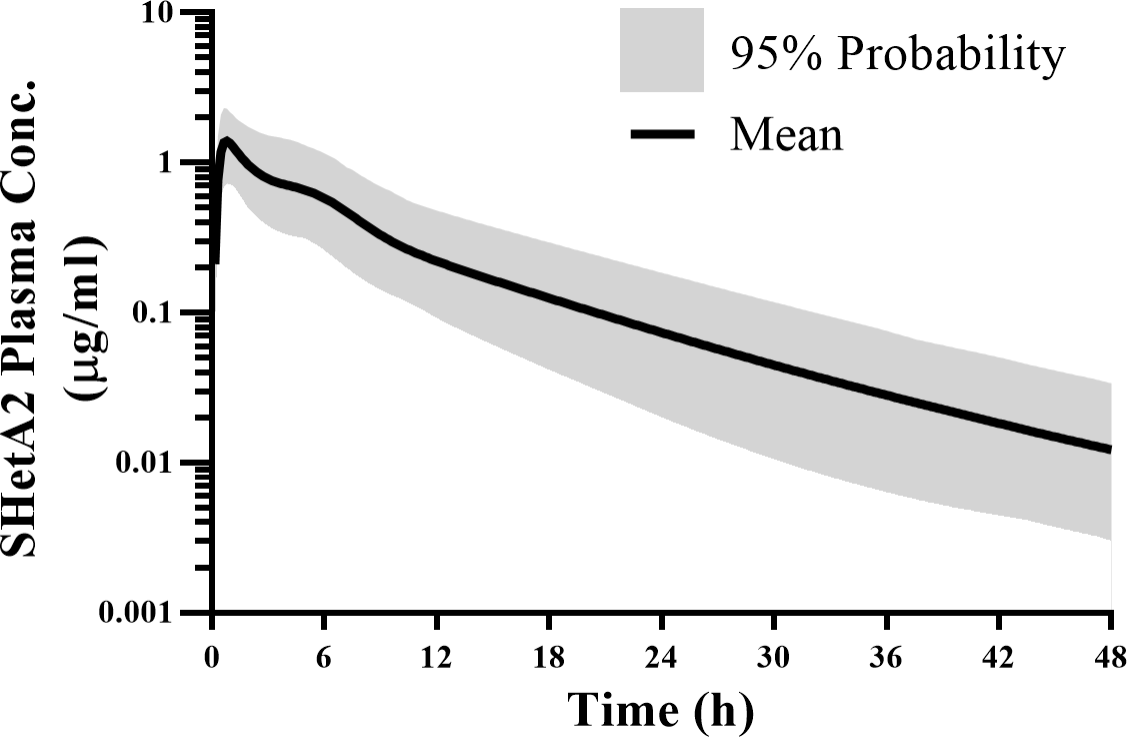
Simulated human PK profile of SHetA2 at 10 mg/kg obtained from 100 virtual subjects in the population simulator of GastroPlus with a two-compartment PK model with physiologically-based gut absorption model for humans. The solid line shows the mean concentrations and the shaded portion shows the 90% probability contour for the spread of concentrations around the mean.

The human oral PK profile (Fig 6) was simulated using GastroPlus with a two-compartment PK model with a physiologically-based gut absorption model for humans. For the disposition parameters, we used the allometrically scaled human PK parameters *V*_*1*_ (36.2 L), *V*_*2*_ (68.5 L), *CL*_*D*_ (15.2 L/h), and MLP-based CL (17.3 L/h) to predict the concentration-time profile of SHetA2 in humans. Evaluation of absorption rate constant (*k*_*A*_) was difficult, as there was no correlation in *k*_*A*_ across different species (Table 3). Thus, instead of a single first-order absorption kinetics, we linked the disposition model with the ACAT model, a physiological gut model available in the GastroPlus program, to predict the drug’s absorption behavior in humans. A clinical trial simulation was run for 100 virtual subjects with doses increasing from 2 mg/kg under the fasted state. The predicted human PK profile (Fig 6) shows rapid absorption and prolonged systemic exposure up to 36–48 h. The mean predicted bioavailability was 18.8% (7.4–42%) which is very close to the maximum bioavailability (18.6%) observed from preclinical species at doses < 100 mg/kg. The prediction also suggested no significant difference in bioavailability from dosing under the fed state (18.1%). Based on the simulations, a *C*_*max*_ of 4 μM could be achieved at a dose of around 10 mg/kg in humans (85-kg body weight).

## Discussion

The lead Flex-Het, SHetA2, was advanced for further clinical development as an anticancer and chemoprevention drug after extensive preclinical evaluations that revealed a broad safety profile and a reasonable in vivo PK profile [11]. In the present study, we demonstrated interspecies scaling and computational modeling of preclinical PK data of SHetA2 from three species (mouse, rat, and dog), and the application of the findings for human PK prediction to guide study design of the planned clinical trials.

Allometric scaling is a well-understood concept for interspecies comparison of the disposition parameters with respect to physiological parameters, such as body weight. This relationship has been reviewed extensively and utilized as an important tool for extrapolation of preclinical data to humans in terms of predicting the time course of drug profiles and determining first-in-human dose [21]. The major route of drug elimination is an important determinant in predicting human CL via different allometric approaches. An in vitro stability study in plasma revealed extensive SHetA2 degradation at physiological temperature with the estimated half-life of 12.7 h across the animal species, e.g., mouse, rat, human [13]. Besides this chemical decomposition in plasma, SHetA2 also appears to undergo hepatic metabolism: studies of in vitro metabolism in rat and human liver microsomes and in vivo metabolism in mice and rats identified hydroxylated SHetA2 metabolites and/or thioester GSH adduct [17]. However, the relative contributions of chemical decomposition and metabolism to total systemic clearance have not been determined. The human clearance predicted from different allometric approaches (e.g., simple allometry and MLP correction) ranged from 17–40 L/h, and the smaller value was used to select the proposed sampling time points (e.g., up to 48 h post-dosing).

The SHetA2 absorption was highly dependent on species, doses, formulation, and possibly other factors. Species-dependent factors, such as gastric and intestinal transit time, blood flow rate, pH, and gut enzyme and transporter distribution can result in differences in the rate and/or extent of drug absorption. Due to the hydrophobic nature of the compound, formulation with different excipients could have played an important role in its oral absorption. Different species also have different dietary habits, which influence the absorption of drugs among species [25].

The absorption rate constant (*k*_*A*_) for oral SHetA2 administration varied across species (0.076–1.1 *h*^*-1*^) and was difficult to correlate for humans, which is not uncommon [26]. In some cases, an inverse relationship of *k*_*A*_ was observed with respect to increasing body weight [27], whereas in other cases, the predicted human *k*_*A*_ was assumed to be the average of the absorption rate constants from preclinical species [28]. The oral absorption kinetics of SHetA2 in mice and rats was well characterized by a simple first-order absorption model, but with quite different *k*_*A*_ values (0.54 vs. 0.076 h^-1^): a relatively fast absorption in mice, but prolonged drug absorption (i.e., flip-flop kinetics) in rats. Such a flip-flop phenomenon may be because of formulation-related factors, considering the different oral formulations used in mouse and rat studies. However, other factors cannot be ruled out, as the flip-flop phenomenon may arise from various physicochemical and physiological mechanisms, including solubility-limited absorption, alteration in membrane permeability, and various anatomical and physiological differences between species [29].

**Fig 7:**
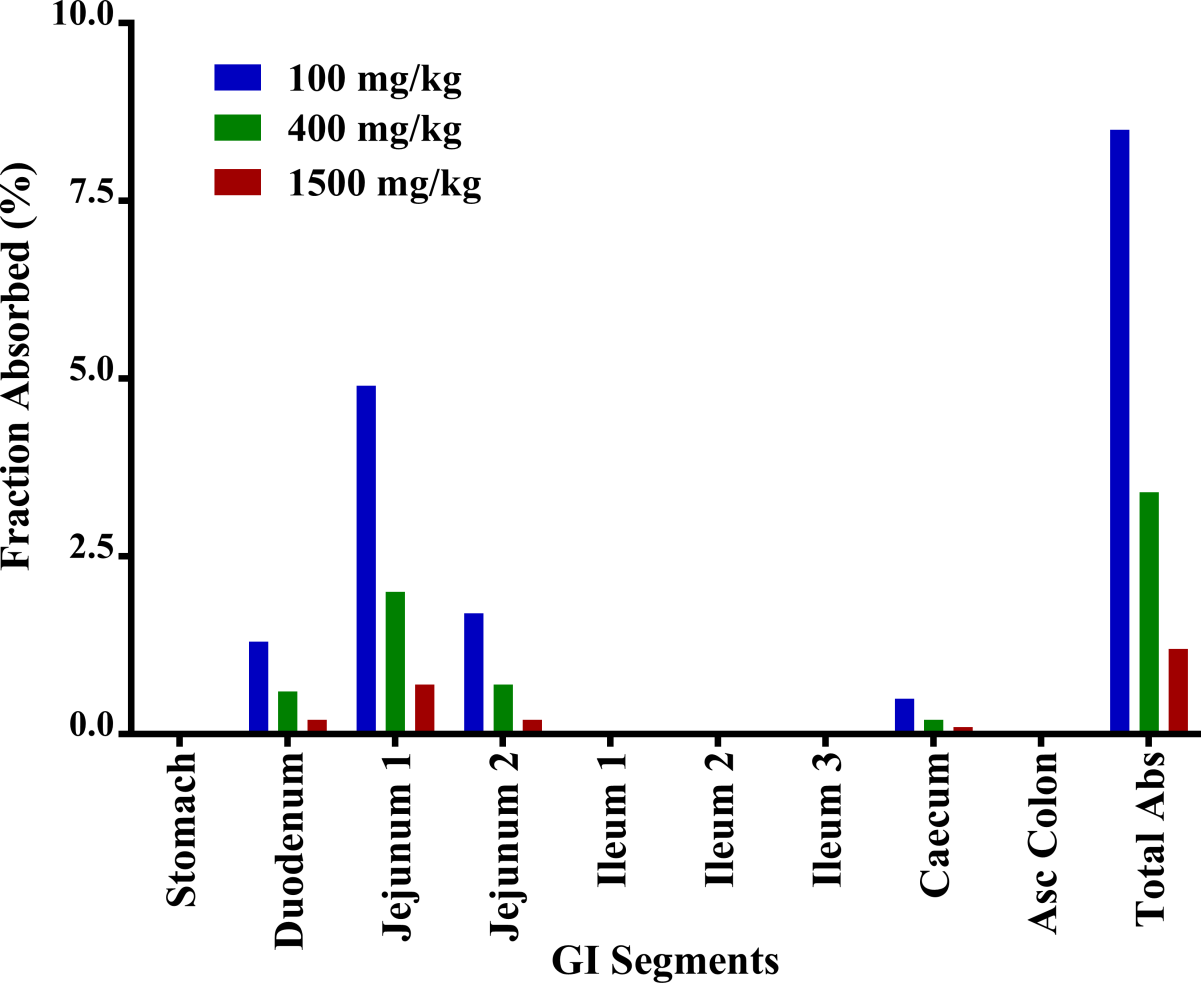
The fraction of regional absorption under fed condition in dogs after a single dose of SHetA2 suspension.

Oral doses in dogs displayed complex concentration-time profiles depending on doses, with double peak phenomenon at high doses. Multiple peaks may arise not only from enterohepatic recirculation, but also by other means, such as absorption from multiple sites [30], reduced gastric motility, absorption differences caused by pH-dependent permeability, transporter densities, and solubility differences due to pH, efflux mechanisms, and the presence of absorption window across the gastrointestinal tract [31]. The double peak could be present due to a reduction in gastric motility caused by its muscle relaxant effect [30]. In our case, enterohepatic recycling was first ruled out as a cause of the double peaks in dogs, because the same phenomenon was not observed after IV administration of SHetA2 [32]. SHetA2 is a weak acid drug with a pKa = 11 [24]. Such drugs (e.g., aspirin, warfarin, and barbiturates) are absorbed much more slowly from the stomach than from the small intestine, and their rates of absorption are directly related to gastric emptying in humans [33]. Thus, based on the physicochemical nature of SHetA2, the absorption in dogs was explained with a gastrointestinal transit absorption model with a major contribution in absorption from initial and later GI segments. To confirm this phenomenon, we analyzed the regional absorption of SHetA2 through different segments of the GI tract using the compartmental absorption and transit model in the GastroPlus for all doses administered in the dog. SHetA2 absorption in different GI regions was estimated by the calculated fraction absorbed. Simulations from the GastroPlus supported our modeling of absorption from initial and later GI segments. SHetA2 was primarily absorbed in the upper GI tract, including the duodenum and jejunum, followed by absorption from the caecum (Fig 7), with almost no absorption from the ileum. In dogs under fed state, the transit time until jejunum and caecum are 2.6 and 7.1 h (GastroPlus), respectively, which correlated closely with the time for the first peak at 1–3 h and delayed absorption until 9 h.

The bioavailability was >10% at doses of up to 100 mg/kg. Upon further dose increases in dogs, however, bioavailability was sharply reduced. This is thought to be due to the limited gut solubility because of the highly hydrophobic nature of the compound; at higher doses, most of the administered drug may be excreted in feces without being absorbed into the systemic circulation. In the case of saturable absorption, therefore, limited oral bioavailability needs to be taken into consideration when calculating human doses from the preclinical toxicity study. Thus, we considered the dose of 100 mg/kg as the new NOAEL at which saturation of drug absorption takes place with no toxicity [22]. Our suggested dose for SHetA2 (2 mg/kg) is much lower than 1/50th of the rat NOAEL (30 mg/kg) that was originally estimated based on the 28-day toxicology studies. Based on our findings, the phase 0 clinical trial is currently planned (IRB#5407) with a starting dose of 2 mg/kg. The first dose will be one capsule containing 170 mg of SHetA2 (e.g., 2 mg/kg corresponding to an 85-kg person). The dose will be escalated by increasing the number of capsules up to a concentration (4 μM) at which target modulation occurs in preclinical models.

Finally, we utilized an empirical allometric approach for prediction of clearance, but further studies are needed to investigate enzymatic metabolic stability quantitatively and involved CYP isoforms for better prediction of potential drug-drug interaction in future clinical studies. The inclusion of a more mechanistic approach, such as the advanced dissolution, absorption, and metabolism (ADAM) model [34], is planned. We are currently working toward the development of a physiologically-based pharmacokinetic (PBPK) model in mice and humans to provide insight into the characteristics of clearance, tissue distribution, and oral absorption of SHetA2.

## Conclusion

The present study characterized preclinical pharmacokinetics of SHetA2 using computational modeling and interspecies scaling. We provided (a) the first PK models for SHetA2 that depicted the disposition and complex absorption kinetics in preclinical species, and (b) computational tools to predict human PK profiles. The findings and computational tools may be used to assist with a better and safe design for the planned clinical trials of SHetA2.
